# Effectiveness of empirical anti-pseudomonal antibiotics for recurrent COPD exacerbation: a multicenter retrospective cohort study

**DOI:** 10.1038/s41598-021-99640-y

**Published:** 2021-10-08

**Authors:** Akihiro Shiroshita, Chisato Miyakoshi, Shunta Tsutsumi, Hiroshi Shiba, Chigusa Shirakawa, Kenya Sato, Shinya Matsushita, Yuya Kimura, Keisuke Tomii, Masahiro Ohgiya, Yuki Kataoka

**Affiliations:** 1 Department of Respiratory Medicine, Ichinomiyanishi Hospital, 1 Kaimeihira, Ichinomiya, Aichi 494-0001 Japan; 2 Johns Hopkins Bloomberg School of Public Health, Baltimore, USA; 3 Systematic Review Workshop Peer Support Group (SRWS-PSG), Osaka, Japan; 4 Department of Research Support, Center for Clinical Research and Innovation, Kobe City Medical Center General Hospital, 2-1-1, Minatojimaminamimachi, Chuo-ku, Kobe, Hyogo 650-004 Japan; 5 General Medicine, Awa Regional Medical Center, Tateyama, Japan; 6 Post Graduate Education Center, Kameda Medical Center, Kamogawa, Japan; 7 Department of Respiratory Medicine, Kobe City Medical Center General Hospital, 2-1-1, Minatojimaminamimachi, Chuo-ku, Kobe, Hyogo 650-004 Japan; 8 Department of Thoracic Medicine, Saiseikai Yokohamashi Tobu Hospital, 3-6-1 Shimosueyoshi Tsurumi Ward, Yokohama, Kanagawa 230-0012 Japan; 9 Center for Pulmonary Diseases, Department of Respiratory Medicine, National Hospital Organization Tokyo National Hospital, 3-1-1 Takeoka, Kiyose-shi, Tokyo, 204-8585 Japan; 10 Department of Internal Medicine, Kyoto Min-Iren Asukai Hospital, Tanaka Asukai-cho 89, Sakyo-ku, Kyoto, 606-8226 Japan; 11 Section of Clinical Epidemiology, Department of Community Medicine, Kyoto University Graduate School of Medicine, Yoshida Konoe-cho, Sakyo-ku, Kyoto, 606-8501 Japan; 12 Department of Healthcare Epidemiology, Kyoto University Graduate School of Medicine/Public Health, Yoshida Konoe-cho, Sakyo-ku, Kyoto, 606-8501 Japan

**Keywords:** Health care, Medical research

## Abstract

Although frequent chronic obstructive pulmonary disease (COPD) exacerbation has been associated with the isolation of *Pseudomonas aeruginosa* (PA) in sputum cultures, it remains unknown whether the empirical use of anti-pseudomonal antibiotics can improve outcomes in patients with frequent COPD exacerbations. This multicenter retrospective cohort study evaluated whether the empirical use of anti-pseudomonal antibiotics improves the length of the hospital stay in patients with recurrent COPD exacerbation (≥ 2 admissions from April 1, 2008 to July 31, 2020). For statistical analysis, a log-linked Gamma model was used. Parameters were estimated using a generalized estimating equation model with an exchangeable correlation structure accounting for repeated observations from a single patient. Covariates included age, body mass index, home oxygen therapy use, respiratory rate, heart rate, oxygen use on admission, mental status, systemic steroid use, activities of daily living, and the number of recurrences. Hospital-specific effects were specified as fixed effects. In total, 344 patients and 965 observations of recurrent COPD exacerbations were selected. Anti-pseudomonal antibiotics were used in 173 patients (18%). The estimated change in the length of the hospital stay between anti-pseudomonal and non-anti-pseudomonal antibiotics groups was 0.039 days [95% confidence interval; − 0.083, 0.162]. Anti-pseudomonal antibiotics could not shorten the length of the hospital stay.

## Introduction

Chronic obstructive pulmonary disease (COPD) is one of the most common respiratory diseases^1^. Patients with COPD frequently experience acute exacerbations; the event rate has been reported as approximately 1–2 times per year^2^. Following COPD exacerbations, background factors such as body mass index, obstruction, dyspnea, and exercise capacity may worsen^3^. Moreover, previous hospitalization for COPD exacerbation has been reported to be an important prognostic factor in patients with COPD exacerbation^4,5^.

Antibiotics are used mainly in patients with moderate or severe exacerbations and/or cough and sputum purulence^6^. Initial empirical treatment with narrow-spectrum antibiotics, such as aminopenicillin with clavulanic acid, macrolide, and tetracycline, is recommended for the entire population with COPD exacerbation, based on previous systematic reviews^7,8^. However, evidence that can guide decisions regarding whether to choose narrow-spectrum or broad-spectrum antibiotics is lacking. Based on expert opinions, local bacterial resistance patterns should be considered^6^. Furthermore, for patients with frequent exacerbations, anti-pseudomonal antibiotic use should be considered, as frequent COPD exacerbations have been reported as associated with the isolation of *Pseudomonas aeruginosa* (PA)^9^. However, it is unclear whether empirical treatment for PA can improve outcomes in patients with frequent COPD exacerbations^10^. Therefore, we aimed to assess the effectiveness of empirical anti-pseudomonal antibiotics in patients with recurrent COPD exacerbation. We hypothesized that anti-pseudomonal antibiotics would improve the length of the hospital stay in patients with frequent COPD exacerbation.

## Methods

### Study design

The study was designed as a multicenter retrospective cohort study across seven acute general hospitals in Japan: Awa Regional Medical Center, Hyogo Prefectural Amagasaki General Medical Center, Ichinomiyanishi Hospital, Kameda Medical Center, Kobe City Medical Center General Hospital, Saiseikai Yokohamashi Tobu Hospital, and Tokyo National Hospital. This study was conducted in accordance with the Declaration of Helsinki^11^. Additionally, this study was approved by the Institutional Review Board (IRB) of each hospital (registration number: 2020008), and the need for written informed consent was waived by the IRB of Ichinomiyanichi Hospital. This article was prepared in accordance with the Strengthening the Reporting of Observational Studies in Epidemiology (STROBE) Statement (Supplementary table 1)^12^.

### Patient selection

The patient enrolment period depended on the storage terms of the electronic medical records in each hospital during the study period of April 1, 2008 to July 31, 2020. Inclusion criteria were age ≥ 40 years and hospital admittance due to COPD exacerbation ≥ two times during the study period. Hospital admittance due to COPD exacerbation was determined by an admission-precipitating diagnosis of COPD exacerbation based on the 10th revision of the International Statistical Classification of Diseases and Related Health Problems (ICD-10) code (ICD-10 code: J44.1). The exclusion criteria were as follows: empirical use of anti-pseudomonal antibiotics for multidrug-resistant PA that are available in Japan (i.e. colistin, polymyxin B, and fosfomycin).

A validation study of the patient selection based on the ICD-10 code (J44.1) was conducted at Ichinomiyanishi Hospital, Kameda Medical Center, Saiseikai Yokohamashi Tobu Hospital, and Tokyo National Hospital during the study period. Patients who were selected according to the above inclusion criteria were reviewed using the clinical charts from a respiratory physician to confirm the diagnosis of COPD exacerbation.

### Data collection

The following patient data were collected from the Diagnosis Procedure Combination database: age, admission date, discharge date, sex, height, weight, activities of daily living as assessed by the Barthel index, comorbidities, tracheal intubation, and prognosis. Other patient data were collected through the review of electrical medical records: baseline COPD stage, type of inhaler used (inhaled corticosteroid, long-acting beta2-agonist, and long-acting muscarinic antagonist), home oxygen therapy use, prior PA isolation, vital signs (systolic blood pressure, respiratory rate, heart rate), mental status, oxygen use on admission, systemic steroid therapy, and antibiotic therapy.

### Outcomes

The primary outcome was the length of the hospital stay. The outcome was measured repeatedly for each patient, and observations were nested within individuals and hospitals. Thus, patients and hospitals were considered as clusters.

### Treatment of interest

The treatment of interest comprised the empirical use of anti-pseudomonal antibiotics on admission or the next day, regardless of the dose and route of administration. The treatment group was defined based on the initial antibiotics, and did not change according to modifications made to these antibiotics after the initial therapy. The anti-pseudomonal antibiotics comprised drugs that are available in Japan are the following: ceftazidime, cefozopran, cefepime, carbapenem (biapenem, doripenem, imipenem, and meropenem), piperacillin, piperacillin/tazobactam, aminoglycoside (gentamicin, tobramycin, and amikacin), quinolone (ciprofloxacin, levofloxacin, garenoxacin, and gatifloxacin), and aztreonam.

### Covariates

Based on the previous literature, the following factors were selected as potential confounding factors: age, body mass index, home oxygen therapy use (binary data), respiratory rate, heart rate (≥ 109 beats/min or not), oxygen use on admission (binary data), mental status (altered mental status or not), systemic steroid use on admission or the next day regardless of the dose (binary data), activities of daily living (Barthel index), and the number of recurrences^4,13–18^.

### Statistical analysis

Patient characteristics are summarized as numbers and percentages for categorical variables and as the median and interquartile range (IQR) for continuous variables. Our statistical analysis is summarized in the GitHub repository^19^. A log-linked Gamma model was used to evaluate the association between each variable and the length of the hospital stay^20^. Since repeated observations were obtained from a single patient, we used the generalized estimating equation method with an exchangeable correlation structure and robust standard error estimation. Hospital-specific effects were implemented as fixed effects.

Missing data were imputed using multiple imputations by chained equations on the assumption that data were missing at random^21^. Covariates of the outcome analysis (number of recurrences, age, COPD stage, home oxygen therapy use, oxygen use on admission, heart rate, respiratory rate, mental status, systemic steroid use, admitting hospital), the treatment variable (anti-pseudomonal antibiotic use), and the outcome variable (length of the hospital stay), in addition to systolic blood pressure as an auxiliary variable, were used to estimate the missing data^22,23^. The results for 100 imputed datasets were aggregated using Rubin’s rule^24^.

As a sensitivity analysis, we constructed a Bayesian model in which both patient- and hospital-specific effects were implemented as random effects using the complete case dataset^25^. This model can be described as follows:

$$ \begin{aligned} & Y_{ijk} \sim\,Gamma\left( {s, s/\mu_{ijk} } \right) \\ & \log \left( {\mu_{ijk} } \right) = X_{ijk} \beta + b_{j} + b_{k} \\ & \sum b_{j} = 0, \sum b_{k} = 0 \\ \end{aligned} $$

where Y_*ijk*_ is the length of the *i*-th stay of patient *j* in hospital *k*, and the mean of the Gamma distribution (log(*μ*) with the shape parameter “*s”*). Besides, log(*μ*) is determined by explanatory variables, *X*, and the patient- and hospital-specific effects with a zero-sum constraint, *b*_*j*_ and *b*_*k*_. Non-informative uniform distribution was used for all parameters. We set four separate sampling chains, each consisting of 5000 samples (including 4000 samples discarded for convergence). We evaluated the sampling convergence by visually inspecting the trace plot and by using the Gelman-Rubin statistic (R-hat), which indicated a good convergence when less than 1.1.

We also conducted a sensitivity analysis incorporating the COPD stage (Stage ≥ III or not), and the inhaler uses (inhaled corticosteroid, long-acting beta2-agonist, and long-acting muscarinic antagonist) into the a log-linked Gamma model. We imputed missing data using the same imputation method as the main analysis and combined the results. All statistical analyses were performed using R software version 4.0.2 (R Foundation for Statistical Computing, Vienna, Austria). For Bayesian model analyses, we used the probabilistic programming language, Stan (Stan Development Team).

## Results

The validation study conducted across four hospitals revealed that patient selection for recurrent COPD exacerbation using the ICD-10 code resulted in 6 of 108 false positives (positive predictive value of 94%).

**Figure 1:**
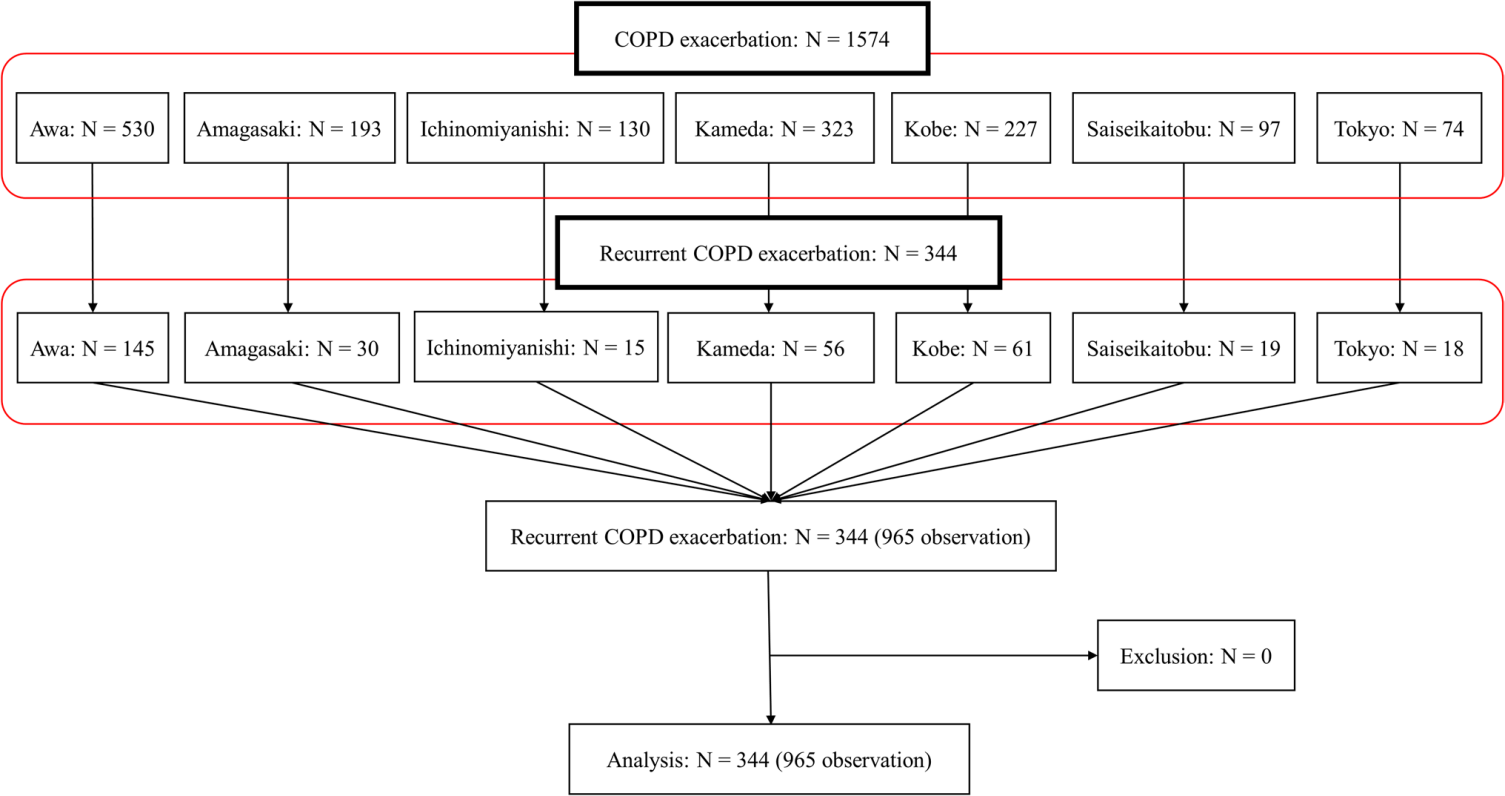
Patient selection flowchart. COPD = chronic obstructive pulmonary disease.

The patient selection flowchart is shown in Fig. 1. Among the selected 1574 patients with COPD exacerbation, 344 (22%) were hospitalized with an admission-precipitating diagnosis of COPD exacerbation ≥ 2 times during the study period (total number of observations/cases: 965). No patients were excluded because of the empirical use of anti-pseudomonal antibiotics for multidrug-resistant PA. The median number of recurrences was 3 (IQR: 2–4), and the median interval to the next hospitalization was 176 days (IQR: 55–507 days). The patients’ characteristics are summarized according to treatment in Table 1.

**Table 1:** Patient characteristics per observation.

Characteristic	Non-anti-pseudomonal antibiotics group (N* = 792, 82%)	Anti-pseudomonal antibiotics group (N = 173, 18%)	Total (N = 965)
**Demographic characteristics**			
Age (years, mean, SD^†^)	78 (9)	80 (8)	78 (9)
Male (number, %)	697 (88)	154 (89)	851 (88)
Height (cm, SD)	160 (11)	161 (8)	160 (10)
Missing data (number, %)	156 (20)	32 (19)	187 (20)
Weight (kg, SD)	53 (12)	52 (12)	52 (12)
Missing data (number, %)	133 (17)	11 (6)	143 (15)
**COPD**^**‡**^** stage**			
I (number, %)	36 (9)	2 (4)	38 (9)
II (number, %)	120 (31)	18 (40)	138 (32)
III (number, %)	155 (40)	15 (33)	170 (39)
IV (number, %)	79 (20)	10 (22)	89 (21)
Missing data (number, %)	402 (51)	128 (74)	530 (55)
Inhaled corticosteroid (number, %)	288 (36)	78 (45)	366 (38)
Long-acting beta2-agonist (number, %)	427 (54)	109 (63)	536 (56)
Long-acting muscarinic antagonist (number, %)	493 (62)	114 (66)	607 (63)
Home oxygen therapy users (number, %)	347 (44)	66 (38)	413 (43)
Activities of daily living score^¶^ (score, IQR^§^)	45 [10–80]	30 [0–55]	45 [5–70]
Missing data (number, %)	128 (16)	25 (15)	13 (1)
Median number of recurrences (number, IQR)	3 [3, 4]	3 [3–5]	3 [2–4]
Median time to next hospitalization (days, IQR)	183 [58–533]	151 [53–424]	176 [55–507]
**Comorbidities**			
Asthma (number, %)	149 (19)	39 (23)	188 (20)
Bronchiectasis	16 (2)	4 (2)	20 (2)
Cancer (number, %)	83 (11)	16 (9)	99 (10)
Diabetes mellitus (number, %)	107 (14)	35 (20)	142 (15)
Heart failure (number, %)	142 (18)	31 (18)	173 (18)
**Vital signs**			
Altered mental status (number, %)	105 (13)	29 (17)	134 (14)
Systolic blood pressure (mmHg, mean, SD)	138 (27)	133 (27)	137 (27)
Missing data (number, %)	41 (5)	2 (1)	43 (4)
Heart rate (beats/min, mean, SD)	99 (20)	105 (22)	100 (20)
Missing data	30 (4)	4 (2)	34 (4)
Respiratory rate (breaths/min, mean, SD)	24 (6)	26 (7)	25 (6)
Missing data (number, %)	62 (8)	18 (10)	80 (8)
Oxygen use on admission (number, %)	604 (76)	140 (81)	744 (77)
Missing data (number, %)	19 (2)	0 (0)	19 (2)
**Treatment**			
Steroid therapy (number, %)	663 (84)	160 (93)	823 (85)
**Prognosis**			
Length of hospital stay (days, IQR)	11 [8–17]	12 [9–21]	12 [8–18]
Tracheal intubation (number, %)	39 (5)	17 (10)	56 (6)
Death (number, %)	37 (5)	18 (10)	55 (6)

*: N = number; †: SD = standard deviation; ‡: COPD = chronic obstructive pulmonary disease; § IQR = interquartile range; ¶: Activities of daily living score is defined as the Barthel index. A high score indicates a higher activity level.

PA was detected in at least 75 of 344 patients (22%) during the study period. The strategies for empirical antibiotic use are summarized in Table 2 and Supplemental Table 2. Piperacillin/tazobactam was used in 70% of anti-pseudomonal antibiotic cases, and ceftriaxone was used in 73% of non-anti-pseudomonal antibiotic cases. Although a sputum sample was not collected in all cases, at least 75 of 965 observations/cases (8%) showed positive sputum-culture results for PA before admission (characterized in Supplemental Table 3). Among these 75 cases, anti-pseudomonal antibiotics were used in 20 (27%). None of the patients received double coverage with anti-pseudomonal antibiotics. Only 13% of cases received antibiotics covering atypical respiratory pathogens, including *Mycoplasma pneumonia*, *Legionella* spp., and *Chlamydia* spp. In the non-anti-pseudomonal antibiotics group, 18 of 474 cases (4%) were switched to anti-pseudomonal antibiotics during hospitalization.

**Table 2:** Empirical antibiotics therapy.

Antibiotics	Number
**Anti-pseudomonal antibiotics (N = 173)**	
Ceftazidime	7
Cefozopran	2
Cefepime	16
Carbapenem	6
Biapenem	0
Doripenem	0
Imipenem	0
Meropenem	6
Piperacillin	4
Piperacillin/tazobactam	120
Aminoglycoside	1
Gentamicin	0
Tobramycin	0
Amikacin	1
Quinolone	13
Ciprofloxacin	1
Levofloxacin	11
Garenoxacin	1
Gatifloxacin	0
Aztreonam	4
**Non-anti-pseudomonal antibiotics (N = 474)**	
Amoxicillin	5
Amoxicillin/clavulanic acid	22
Ampicillin	4
Cefuroxime	1
Penicillin-G	2
Sulbactam/ampicillin	56
Cefotiam	4
Cefotaxime	2
Ceftriaxone	345
Vancomycin	11
Sulfamethoxazole/trimethoprim	4
Macrolide	64
Azithromycin	46
Clarithromycin	1
Erythromycin	17
Tetracycline	7
Minomycin	6
Doxycycline	1
Moxifloxacin	1

The main analysis with multiple imputations and the generalized estimating equation revealed that the estimated change in the length of the hospital stay between the anti-pseudomonal and non-anti-pseudomonal antibiotics groups was 0.039 days [95% confidence interval; − 0.083, 0.162] (Table 3). This non-significant result was consistent with the results of the sensitivity analyses.

**Table 3:** The estimated difference in the length of the hospital stay between the anti-pseudomonal and non-anti-pseudomonal antibiotics groups.

Statistical model	Coefficient	95% confidence interval or 95% highest density interval
Multiple imputations and the generalized estimating equation with an exchangeable correlation structure	0.039	− 0.083, 0.162
A complete case analysis and three-level Bayesian hierarchical model	0.178	− 1.836, 2.060
Multiple imputations and the generalized estimating equation with additional variables of COPD stage and inhaler uses	0.036	− 0.085, 0.157

*COPD* Chronic obstructive pulmonary disease.

## Discussion

This multicenter retrospective cohort study aimed to evaluate the effectiveness of empirical anti-pseudomonal antibiotics in terms of the length of the hospital stay in patients with frequent COPD exacerbation. We hypothesized that anti-pseudomonal antibiotics would reduce the length of the hospital stay. However, after adjusting for known confounding factors, the length of the hospital stay was not significantly different between the non-anti-pseudomonal and anti-pseudomonal antibiotics groups.

Although the PA detection rate in the present study was not lower than that in previous studies conducted in other countries (22% in the present study vs 4–13%), our hypothesis was not supported^10,26^. To the best of our knowledge, this study is the first to assess the effectiveness of anti-pseudomonal antibiotics in patients with recurrent COPD exacerbation. Our non-significant results are consistent with a previous prospective cohort study targeting hospitalized patients who had a positive culture result for PA in lower-tract specimens^27^. In this previous study, only 18% of patients were treated with anti-pseudomonal antibiotics and, after adjusting for confounding factors, inadequate initial antibiotic use was not associated with 12-month mortality. In contrast, inappropriate initial antibiotic use in the context of PA pneumonia has been reported as associated with increased 28-day mortality^28^. This difference in results may be due to differences in the proportion of patients with pneumonia. In a study of PA pneumonia, the 28-day mortality rate was 51%, which is comparable to that in other reports^29^. Although PA detection among patients with COPD has been reported as associated with extremely high 2-year all-cause mortality (23–41%), the 1-month mortality was 0%^10,30^. Considering the low 1-month mortality in patients with COPD exacerbation with PA isolation, empirical anti-pseudomonal antibiotics may not have adequate power to change the prognosis during hospitalization.

Initial treatment with non-anti-pseudomonal antibiotics may spare anti-pseudomonal antibiotic use. The median time to the next hospitalization was approximately half a year, and the previous use of antibiotics was detected in only one-third of observations. In the present study, although at least 70 observations/cases had positive sputum-culture results for PA before admission, PA was covered by empirical antibiotics in only 29% of cases. The patient characteristics and length of the hospital stay were not substantially different between anti-pseudomonal and non-anti-pseudomonal antibiotics groups. Furthermore, a change in the treatment from non-anti-pseudomonal antibiotics to anti-pseudomonal antibiotics occurred in only 4% of cases. Currently, multidrug-resistant organisms are a global concern^31^. The judicious use of broad-spectrum antibiotics can reduce the number of drug-resistant microorganisms^32,33^. Additionally, the use of broad-spectrum antibiotics is associated with a high cost of care and increased antibiotic-associated side effects, such as *Clostridium difficile* infection^34^. Therefore, a strategy of empirical narrow-spectrum antibiotic use in patients with recurrent COPD exacerbation may be an acceptable choice.

This multicenter retrospective study has numerous strengths. First, this study was based on daily clinical practice. In contrast to the GOLD 2020 report and a network meta-analysis of randomised controlled trials (RCTs), the antibiotics used in the present cohort were mainly intravenous antibiotics^6,35^. This may be due to the large number of elderly patients requiring some assistance; the average age of the present cohort was 80 years, and only 175 of 965 (18%) cases were fully independent. The present study results may better reflect hospitalized patients in daily practice rather than selected patients in RCTs^8^. Second, the number of observations was large compared to that in previous RCTs. The number of included patients/observations was much larger than that in a meta-analysis of four RCTs assessing the effect of currently used antibiotics on the length of the hospital stay (965 observations in the present study vs 393)^8^. Furthermore, an ongoing RCT targeting patients with at least one hospitalization within the prior 12 months and the detection of PA in a sputum culture has an expected total sample size of 150 (for the comparison of the anti-pseudomonal antibiotics group and the placebo group)^36^, which is far smaller than that in the present study. Thus, the present results may fill gaps not covered by these RCTs.

The main caveat regarding this study is that we only evaluated the length of the hospital stay. Although the length of the hospital stay is associated with the survival of hospitalized patients with COPD, as an outcome, it is short-term and soft. Based on the results of previous systematic reviews, hard outcomes such as in-hospital death and 30-day mortality could not be evaluated in the present study because of its sample size^7,8^. Moreover, the time to the next exacerbation could not be evaluated because of unmeasured confounding factors, such as post-admission baseline treatments for COPD. In a previous retrospective cohort study with unmeasured confounding factors, antibiotic use was associated with improvements in the long-term mortality and time to the next exacerbation^37^. Further large-scale studies are warranted to evaluate hard short-term and long-term outcomes^38^.

The present study had several other limitations. First, although our validation study showed a high predictive value for our patient selection strategy, the number of patients with COPD exacerbation was relatively small, considering that all of the hospitals were acute care and educational hospitals. Patient selection based on the ICD-10 code could have low sensitivity, and there could be many patients who should have been included in this study. In Japan, especially in our hospitals, payment is based on Diagnosis Procedure Combination: a system that reimburses hospitals based on the diagnosis code of hospitalized patients. Patients with a long length of stay or complications during hospitalization might be coded with diagnoses other than COPD exacerbation. Thus, the length of stay may have been right-truncated, and the effect of anti-pseudomonal antibiotics may have been skewed toward the null. Second, the interval between COPD exacerbations was not taken into account, and the variance correlation of the length of the hospital stay within each patient over multiple visits may differ from our expectations. We coped with this issue by using robust variances. Third, there may have been additional confounding factors. For example, although the number of patients with bronchiectasis was not different between both groups, it could be underestimated because bronchiectasis was detected based on ICD-10 (J47). In addition, we could not determine whether the PA isolation was regarded as colonization or pathogenic. We were unable to collect data regarding the other potential pathogens and the susceptibility results, dose of antibiotics, and the duration of COPD. Thus, we may not fully understand the relationship between clinicians’ decision-making process and patients’ outcomes. Further RCTs are needed to address unknown confounding factors. Forth, while we performed a sensitivity analysis using the COPD stage as a confounding factor, there was a substantial amount of missing data. In addition, we collected the information about the COPD Stage from the electronic medical records, and the data reliability might be low because physicians might not write it accurately.

## Conclusions

The results of the present study suggest that empirical anti-pseudomonal antibiotics do not decrease the length of the hospital stay. However, further studies with larger sample sizes are needed to evaluate the effectiveness of anti-pseudomonal antibiotics more precisely.

## Supplementary Information

Supplementary Information.

## Data Availability

The datasets generated and/or analyzed during the current study are not publicly available due to the privacy issues but are available from the corresponding author on reasonable request.
