# Supplementary material for: Effectiveness of empirical anti-pseudomonal antibiotics for recurrent COPD exacerbation: a multicenter retrospective cohort study
Source: Sci Rep. 2021 Oct 8;11:20066. doi: 10.1038/s41598-021-99640-y (PMC8501095; doi:10.1038/s41598-021-99640-y)
Supplement: Supplementary file 1 — Supplementary Information. [file 41598_2021_99640_MOESM1_ESM.docx]

**Effectiveness of empirical anti-pseudomonal antibiotics for recurrent COPD exacerbation: A multicenter retrospective cohort study**

Akihiro Shiroshita*^1,2,3^, Chisato Miyakoshi^4^, Shunta Tsutsumi^5^, Hiroshi Shiba^6^, Chigusa Shirakawa^7^, Kenya Sato^8^, Shinya Matsushita^8^, Yuya Kimura^9^, Keisuke Tomii^7^, Masahiro Ohgiya^9^, Yuki Kataoka^3,10,11,12^

^1^Department of Respiratory Medicine, Ichinomiyanishi Hospital, Ichinomiya, 1 Kaimeihira Ichinomiya, Aichi 491-0001, Japan

^2^Johns Hopkins Bloomberg School of Public Health, Baltimore, USA

^3^Systematic Review Workshop Peer Support Group (SRWS-PSG), Japan

^4^Department of Research Support, Center for Clinical Research and Innovation, Kobe City Medical Center General Hospital, 2-1-1, Minatojimaminamimachi, Chuo-ku, Kobe, Hyogo 650-004, Japan

^5^General Medicine, Awa Regional Medical Center, Tateyama, Japan

^6^Post Graduate Education Center, Kameda Medical Center, Kamogawa, Japan

^7^Department of Respiratory Medicine, Kobe City Medical Center General Hospital, 2-1-1, Minatojimaminamimachi, Chuo-ku, Kobe, Hyogo 650-004, Japan

Japan

^8^Department of Thoracic Medicine, Saiseikai Yokohamashi Tobu Hospital, 3-6-1 Shimosueyoshi Tsurumi Ward, Yokohama, Kanagawa 230-0012, Japan

^9^Center for Pulmonary Diseases, Department of Respiratory Medicine, National Hospital Organization Tokyo National Hospital, 3-1-1 Takeoka, Kiyose-shi, Tokyo 204-8585, Japan

^10^Department of Internal Medicine, Kyoto Min-Iren Asukai Hospital, Tanaka Asukai-cho 89, Sakyo-ku, Kyoto 606-8226, Japan

^11^Section of Clinical Epidemiology, Department of Community Medicine, Kyoto University Graduate School of Medicine, Yoshida Konoe-cho, Sakyo-ku, Kyoto 606-8501, Japan

^12^Department of Healthcare Epidemiology, Kyoto University Graduate School of Medicine / Public Health, Yoshida Konoe-cho, Sakyo-ku, Kyoto 606-8501, Japan

***Corresponding author**:

Akihiro Shiroshita

Department of Respiratory Medicine, Ichinomiyanishi Hospital

1 Kaimeihira, Ichinomiya, Aichi 494-0001, Japan

E-mail: [akihirokun8@gmail.com](mailto:akihirokun8@gmail.com)

ORCID: <https://orcid.org/0000-0003-0262-459X>

**Supplementary tables**

**Supplementary Table 1:** The Strengthening the Reporting of Observational Studies in Epidemiology (STROBE) Checklist

|  | Item No | Recommendation | Page No |
| --- | --- | --- | --- |
| **Title and abstract** | 1 | (*a*) Indicate the study’s design with a commonly used term in the title or the abstract | 1 |
|  |  | (*b*) Provide in the abstract an informative and balanced summary of what was done and what was found | 3 |
| Introduction | | | |
| Background/rationale | 2 | Explain the scientific background and rationale for the investigation being reported | 3-4 |
| Objectives | 3 | State specific objectives, including any prespecified hypotheses | 3-4 |
| Methods | | | |
| Study design | 4 | Present key elements of study design early in the paper | 4 |
| Setting | 5 | Describe the setting, locations, and relevant dates, including periods of recruitment, exposure, follow-up, and data collection | 4 |
| Participants | 6 | (*a*) Give the eligibility criteria, and the sources and methods of selection of participants. Describe methods of follow-up | 5 |
|  |  | (*b*) For matched studies, give matching criteria and number of exposed and unexposed | Not applicable |
| Variables | 7 | Clearly define all outcomes, exposures, predictors, potential confounders, and effect modifiers. Give diagnostic criteria, if applicable | 5-6 |
| Data sources/ measurement | 8* | For each variable of interest, give sources of data and details of methods of assessment (measurement). Describe comparability of assessment methods if there is more than one group | 5-6 |
| Bias | 9 | Describe any efforts to address potential sources of bias | 5-7 |
| Study size | 10 | Explain how the study size was arrived at | 5 |
| Quantitative variables | 11 | Explain how quantitative variables were handled in the analyses. If applicable, describe which groupings were chosen and why | 5-7 |
| Statistical methods | 12 | (*a*) Describe all statistical methods, including those used to control for confounding | 6-7 |
|  |  | (*b*) Describe any methods used to examine subgroups and interactions | Not applicable |
|  |  | (*c*) Explain how missing data were addressed | 6-7 |
|  |  | (*d*) If applicable, explain how loss to follow-up was addressed | Not applicable |
|  |  | (*e*) Describe any sensitivity analyses | 6-7 |
| Results | | |  |
| Participants | 13* | (a) Report numbers of individuals at each stage of study—eg numbers potentially eligible, examined for eligibility, confirmed eligible, included in the study, completing follow-up, and analysed | 7-8, Figure 1 |
|  |  | (b) Give reasons for non-participation at each stage | 7-8, Figure 1 |
|  |  | (c) Consider use of a flow diagram | Figure 1 |
| Descriptive data | 14* | (a) Give characteristics of study participants (eg demographic, clinical, social) and information on exposures and potential confounders | 7-8, Table 1 |
|  |  | (b) Indicate number of participants with missing data for each variable of interest | Table 1 |
|  |  | (c) Summarise follow-up time (eg, average and total amount) | 8 |
| Outcome data | 15* | Report numbers of outcome events or summary measures over time | 8 |

| Main results | 16 | (*a*) Give unadjusted estimates and, if applicable, confounder-adjusted estimates and their precision (eg, 95% confidence interval). Make clear which confounders were adjusted for and why they were included | 8, Table 3 |
| --- | --- | --- | --- |
|  |  | (*b*) Report category boundaries when continuous variables were categorized | 6 |
|  |  | (*c*) If relevant, consider translating estimates of relative risk into absolute risk for a meaningful time period | Not applicable |
| Other analyses | 17 | Report other analyses done—eg analyses of subgroups and interactions, and sensitivity analyses | 11 |
| Discussion | | | |
| Key results | 18 | Summarise key results with reference to study objectives | 8-9 |
| Limitations | 19 | Discuss limitations of the study, taking into account sources of potential bias or imprecision. Discuss both direction and magnitude of any potential bias | 10-12 |
| Interpretation | 20 | Give a cautious overall interpretation of results considering objectives, limitations, multiplicity of analyses, results from similar studies, and other relevant evidence | 8-12 |
| Generalisability | 21 | Discuss the generalisability (external validity) of the study results | 11,12 |
| Other information | | | |
| Funding | 22 | Give the source of funding and the role of the funders for the present study and, if applicable, for the original study on which the present article is based | 18 |

**Supplementary Table 2:** Previous use of antibiotics for each hospitalization

|  | Previous use of any antibiotics (number, %) | No previous use of any antibiotics (number, %) |
| --- | --- | --- |
| Any antibiotic use on hospitalization (number, %) | 106 (17) | 85 (14) |
| No antibiotic use on hospitalization (number, %) | 110 (18) | 320 (52) |
|  | Previous use of any anti-pseudomonal antibiotics (number, %) | No previous use of any anti-pseudomonal antibiotics (number, %) |
| Any anti-pseudomonal antibiotic use on hospitalization (number, %) | 441 (71) | 75 (12) |
| Non-anti-pseudomonal antibiotic use on hospitalization (number, %) | 64 (10) | 41 (7) |

**Supplementary Table 3:** Characteristics of patients with positive sputum-culture results for *Pseudomonas aeruginosa* before admission

| Characteristics | Non-anti-pseudomonal antibiotics group (N^*^ = 55, 73%) | Anti-pseudomonal antibiotics group (N = 20, 27%) |
| --- | --- | --- |
| Age (years, mean, SD^†^) | 77 (8) | 80 (6) |
| Male (number, %) | 55 (100) | 16 (80) |
| Height (cm, SD) | 164 (8) | 162 (9) |
| Weight (kg, SD) | 58 (16) | 49 (14) |
| COPD^‡^ Stage |  |  |
| Ⅰ (number, %) | 3 (9) | 0 (0) |
| Ⅱ (number, %) | 4 (12.5) | 2 (33) |
| Ⅲ (number, %) | 20 (63) | 2 (33) |
| Ⅳ (number, %) | 5 (16) | 2 (33) |
| Home oxygen therapy use (number, %) | 35 (64) | 10 (50) |
| Activities of daily living score^¶^ (score, IQR^§^) | 35 [9–72] | 25 [8–43] |
| Median number of recurrences (number, IQR) | 4 [3–6] | 3 [3–4] |
| Median time to next hospitalization (days, IQR) | 117 [53–350] | 106 [63–246] |
| Altered mental status (number, %) | 7 (13) | 5 (25) |
| Heart rate (beats/min, mean, SD) | 105 (21) | 111 (18) |
| Respiratory rate (breaths/min, mean, SD) | 25 (6) | 28 (7) |
| Oxygen use on admission (number, %) | 50 (91) | 18 (90) |
| Steroid therapy (number, %) | 45 (6) | 19 (95) |
| Length of hospital stay (days, IQR) | 14 [9–18] | 14 [11–21] |
| Tracheal intubation (number, %) | 4 (0) | 0 (0) |
| Death (number, %) | 4 (7) | 2 (10) |

**Abbreviations**: *: N = number; †: SD = standard deviation; ‡: COPD = chronic obstructive pulmonary disease; § IQR = interquartile range

**Note:** Patients with no missing data are summarized in e-Table 3.

¶: Activities of daily living score is defined as the Barthel index. A high score indicates a higher activity level.
